# Biomaterial Strategies for Selective Immune Tolerance: Advances and Gaps

**DOI:** 10.1002/advs.202205105

**Published:** 2023-01-13

**Authors:** Sean T. Carey, Christopher Bridgeman, Christopher M. Jewell

**Affiliations:** ^1^ University of Maryland Fischell Department of Bioengineering University of Maryland College Park MD 20742 USA; ^2^ US Department of Veterans Affairs VA Maryland Health Care System Baltimore MD 21201 USA; ^3^ Robert E. Fischell Institute for Biomedical Devices College Park MD 20742 USA; ^4^ Department of Microbiology and Immunology University of Maryland Medical School Baltimore MD 21201 USA; ^5^ Marlene and Stewart Greenebaum Cancer Center Baltimore MD 21201 USA

**Keywords:** autoimmunity and allergies, clinical, tolerance, immunology, nanotechnology

## Abstract

Autoimmunity and allergies affect a large number of people across the globe. Current approaches to these diseases target cell types and pathways that drive disease, but these approaches are not cures and cannot differentiate between healthy cells and disease‐causing cells. New immunotherapies that induce potent and selective antigen‐specific tolerance is a transformative goal of emerging treatments for autoimmunity and serious allergies. These approaches offer the potential of halting—or even reversing—disease, without immunosuppressive side effects. However, translating successful induction of tolerance to patients is unsuccessful. Biomaterials offer strategies to direct and maximize immunological mechanisms of tolerance through unique capabilities such as codelivery of small molecules or signaling molecules, controlling signal density in key immune tissues, and targeting. While a growing body of work in this area demonstrates success in preclinical animal models, these therapies are only recently being evaluated in human trials. This review will highlight the most recent advances in the use of materials to achieve antigen‐specific tolerance and provide commentary on the current state of the clinical development of these technologies.

## Graphical abstract

Immunotherapies that induce antigen‐specific tolerance are a transformative goal of emerging treatments for autoimmunity and allergies. Such approaches can halt or reverse disease without immunosuppressive side effects. Biomaterials offer capabilities to drive potent and selective tolerance, including codelivery of small molecules or biologics, immune tissue targeting, and intrinsic immune modulatory profiles conferred by defined materials property and signal display densities.

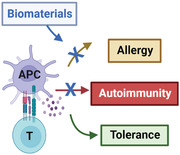

## 1 Introduction

### 1.1 Autoimmunity and Allergy Require More Targeted Therapies

Globally, ≈3–5% of people are affected by autoimmunity, and an additional 10–30% have a form of allergy.^[^ ^1^, ^2^ ^]^ Autoimmunity occurs when the immune system mistakenly attacks self‐molecules—antigens—as foreign, resulting in tissue destruction and pathology. Similarly, allergy occurs when the immune system initiates a disproportionate reaction to a generally innocuous foreign molecule—an allergen. In each situation, the immune system recognizes these antigen and allergen molecules, responding as if they originate from a pathogen, resulting in damaging inflammatory mechanisms.^[^ ^3^ ^]^

While the precise causes of autoimmunity and allergy are not fully elucidated, there are several known risk factors. For autoimmunity, risk factors include human leukocyte antigen (HLA) genotypes, biological sex, and infection. HLA genotype determines antigen presentation machinery in humans. HLA genes with increased autoimmunity risk are hypothesized to result in altered self‐antigen and major histocompatibility complex (MHC) presentation to adaptive immune cells, resulting in inflammation rather than nonreactivity.^[^ ^4^ ^]^ Biological sex is another variable, with a higher prevalence of autoimmunity in females compared to males. This is likely due to the sexual dimorphism of immune responses, wherein males are more susceptible to infections: the stronger immune responses mounted by females may also result in more frequent autoimmunity.^[^ ^5^, ^6^ ^]^ Finally, there is a hypothesized link between certain infections and autoimmunity. Pathogens in these infections have molecular signatures that resemble self‐antigens, thus the resulting immune response can result in targeting of self‐tissue.^[^ ^7^ ^]^ One recent example is a correlative study showing a high association of multiple sclerosis (MS) with Epstein–Barr Virus.^[^ ^8^ ^]^

A number of risk factors are also associated with allergies. Genetically, a range of genes have been showed through genome‐wide association to act as risk factors for autoimmunity.^[^ ^9^ ^]^ Some of these genes include HLA genotypes, as in the case of autoimmunity described above, as well as genes for different immune mediators such cytokine receptors. Allergies also have environmental risk factors, including exposure to antigens, diet, and the make‐up of the gut microbiome.^[^ ^10^ ^]^ In particular, the gut microbiome can change interactions between the mucosal immunity of the gut and the innocuous food allergens encountered in the gastrointestinal tract.^[^ ^11^ ^]^ The intersection of both genetic risk factors and environmental risks likely play strong roles in development of disease.

Current treatments for allergy and autoimmunity rely on varying levels of nonspecific immune suppression using drugs such as steroids or mTOR inhibitors^[^ ^12^ ^]^—however, these approaches are not curative, require lifelong management, and can leave patients immunocompromised or cause susceptibility to serious infections. More recent strategies focus on targeting particular cell types and cytokines using monoclonal antibodies, such as B cell targeting antibodies for MS^[^ ^13^ ^]^ or anti‐tumor necrosis factor (TNF) antibodies for rheumatoid arthritis.^[^ ^14^ ^]^ However, even these highly targeted monoclonal therapies do not differentiate between disease‐causing and healthy immune cells, potentially reducing the immune system's capability to fight infectious disease.

### 1.2 Antigen‐Specific Tolerance is a Therapeutic Goal

**Figure 1:**
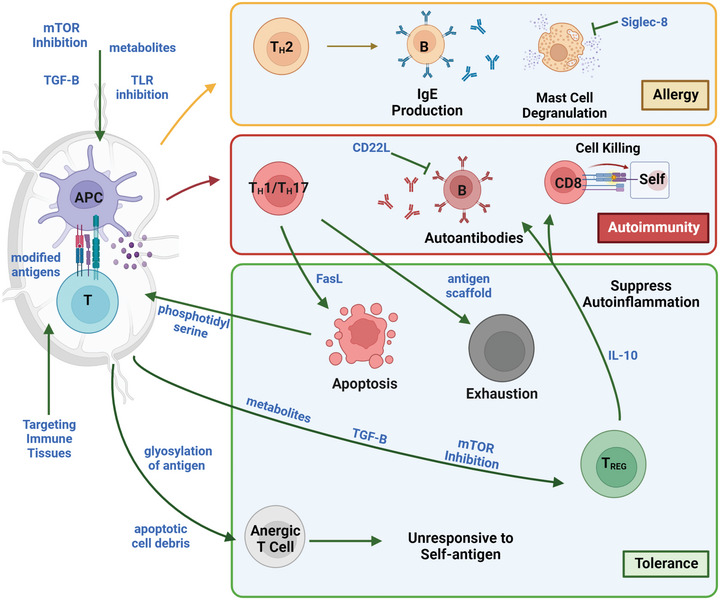
Overview of allergy, autoimmunity, and approaches exploited by biomaterial approaches. APCs present antigen with costimulation and cytokines in lymph nodes (left) to initiate immune responses. Allergic responses (yellow) are driven by T_H_2 cells, B cells producing IgE, and innate immune cells such as mast cells that produce granules such as histamines. Autoimmunity (red) occurs largely via T_H_1 and T_H_17 cells driving tissue destruction via CD8 cytotoxic cells and the production of autoantibodies. Tolerance (green) can be mediated through anergy, apoptosis, and exhaustion of self‐reactive T cells, and T_REG_ that suppress autoimmune responses. Pathways and mechanisms used by materials targeting each scenario are in blue. Created using Biorender.com.

To be “seen” by the immune system, antigens must reach important tissues called lymph nodes—achieved either via passive drainage through lymphatics or by trafficking via phagocytic cells. Once in lymph nodes, specialized antigen‐presenting cells (APCs) present antigen on MHC molecules to activate B and T lymphocytes with receptors that are specific for the antigen. Having recognized their cognate antigen, lymphocytes proliferate and produce antibodies (B cells), organize and recruit other immune cells (CD4 Helper T cells), and kill infected cells (CD8 Cytotoxic T cells).^[^ ^15^ ^]^ During autoimmunity, this process occurs with a self‐antigen instead of a pathogen, resulting in destruction of host tissue via inflammatory T cells subsets (T_H_1, T_H_17) and production of autoantibodies via B cells. In allergy, an innocuous allergen elicits a T_H_2 immune response that recruits immune cells such as mast cells and promotes the production of IgE antibody from B cells. Recognition of allergen by IgE bound to mast cells results in degranulation of effectors such as histamines that cause an allergic response (**Figure** **1**).

In healthy individuals, there are immunological mechanisms that prevent self‐molecules and innocuous foreign antigens from immune targeting—this is termed tolerance. Tolerance includes several different mechanisms working together that maintain immune homeostasis and restore this balance after infection. During the development of immune cells, B and T cells with strong specificities for self‐tissue are deleted to prevent future attack of self‐tissue. Additional critical cell populations include regulatory populations of T cells and B cells termed T_REG_ and B_REG_. These “peripheral” regulatory cell types suppress inflammation toward their antigen specifically rather than promoting inflammation. Regulatory cells achieve this suppression using mechanisms including regulatory cytokine secretion and contact‐based inhibition.^[^ ^16^, ^17^, ^18^ ^]^ In noninflammatory conditions, presentation of antigen to immune cells can drive anergy, a process in which antigen‐specific cells become unresponsive to repeat stimulus with the antigen they recognize. When functional, these processes maintain tolerance through deletion of autoreactive cells, suppression of responses toward self‐antigen, and nonresponsiveness to self‐antigen. The specificity of immune tolerance contrasts with the broader, more transient effects of immune suppression and anti‐inflammatory approaches. During autoimmunity and allergy, immune tolerance toward self or innocuous antigen breaks down, and these antigens are attacked rather than protected or ignored (Figure 1).^[^ ^3^ ^]^ The specificity of the immune responses to self‐antigens and allergens has driven massive interest in antigen‐specific immunotherapies for autoimmunity and allergy to achieve effective and specific immune tolerance. The goal of this strategy is to modify the dysfunctional pathologic immune response toward a particular antigen (i.e., self, allergen), while leaving intact the normal immune response mechanisms required to combat infections. While desirable, the development of antigen‐specific tolerance requires precise spatial and temporal coordination of immune signals—a challenge that biomaterials are well‐suited to address. Thus far, the only clinical successes in antigen‐specific tolerance are in specific immunotherapies (SIT) for mild allergies—which while an important success underscores the allergy and autoimmune conditions that yet lack clinical tolerance approaches.

### 1.3 Biomaterials Offer Greater Precision for Applications in Immune Tolerance

As described above, immune responses and the induction of tolerance are dependent on combinations of signals being integrated over precise timescales and locations for successful outcomes. Additionally, many signals that promote differentiation of tolerizing cell populations can be immunosuppressive if delivered systemically—for example, rapamycin is a potent T_REG_ inducer but can also be an immunosuppressant when delivered systemically.^[^ ^19^ ^]^ Thus, control over how and where signals are encountered is critical for effective tolerizing immunotherapy. Biomaterials offer tunable properties that allow for control over many of the variables that must be controlled for tolerance. These properties include codelivery of antigen and regulatory cues—ensuring both signals are encountered by target cells and tissues to drive the desired regulatory outcome, protection of immunological cargo to prevent degradation and clearance, and targeting of specific immunological tissues or cell populations via manipulation of material stiffness, size, and conjugation with targeting ligand.^[^ ^20^ ^]^ Additionally, biomaterials enable controlled release of components to drive sustained concentrations of immune cargo over time to drive more potent responses, while mitigating potentially toxic concentrations from treatment boluses. While mitigating potential spikes in concentration, sustained exposure to these molecules could drive unwanted effects such as antidrug antibodies which would need to be assessed for clinical applications. The properties of biomaterials enable targeting of the very mechanisms of tolerance described in Section 1.2. For example, surface conjugation of ligands to a particle may be ideal for targeting a cell surface receptor, alternatively a controlled release system may be desirable to drive prolonged response or condition a tissue. Additionally, the particular immune response being corrected may require a customized tolerance approach that materials would enable such as through targeting a pathogenic cell type or delivering a particular payload. Here we focus on the immunological mechanisms that biomaterials are being explored to exploit in the generation of antigen‐specific immunotherapies in allergy and autoimmunity (Figure 1). These areas include modulation using small molecules with and without antigen (Section 2), presentation of biologics such as tolerizing cytokines (Section 3), direct modification of the material properties of antigens (Section 4), targeting tolerizing tissues and cells (Section 5), and exploiting the innate immunomodulatory effect of antigen delivery in particulate or other structures (Section 6). Additionally, we discuss the clinical outlook for antigen‐specific tolerance and progress in the clinical development of biomaterials for this application (Section 7). This review focuses on advances in the last 3 years, as well as select key papers that have advanced the field. We focus on how materials strategies uniquely enable targeting of the complex mechanisms of tolerance and how the biology of tolerance informs material design. While papers for review were selected based on a scope of antigen‐specific tolerance, we pay attention to both the goal of each approach, as well as a critical analysis of how effectively tolerance was demonstrated. In particular, effective demonstration of both the antigen‐specificity of the response as well as durable protection against pathology will be examined. Many approaches in actuality demonstrate transient immunosuppression, rather than tolerance, highlighting an opportunity for future work in the biomaterials fields across a variety of materials technologies. For reviews focused on material properties and design, readers are directed to other reviews.^[^ ^21^, ^22^, ^23^, ^24^, ^25^ ^]^

## 2 Materials Focus and Enhance Tolerizing Effects of Small Molecule Drugs and Metabolites

Many therapeutics for autoimmunity and allergy are small molecule drugs and inhibitors that target pathways of immune activation and inflammation. When delivered systemically, these drugs often have broader or global effects such as immune suppression and off‐target effects on nonimmune cells. In this section, we discuss the unique advantages biomaterials have for enhancing the immunomodulatory effects of small molecule drugs for immune tolerance. These include codelivery of antigen and small molecules, shifting drug profiles from suppressive to tolerizing, and reducing off target effects. Codelivery and coencapsulation of antigen and small molecule ensures that these cues are encountered in the same environment—necessary for more efficient tolerance induction—whereas delivering soluble cues would not ensure delivery to the same target cells. Additionally, the targeting offered by biomaterials allows otherwise toxic or broadly acting drugs to have a more targeted effect, including immunosuppressives such as mTOR inhibitors, metabolic modulators, and even steroids^[^ ^26^ ^]^ and anticancer drugs.^[^ ^27^ ^]^ An advantage of biomaterial delivery of small molecule drugs is to reduce off‐target effects by protecting small molecule cargo via encapsulation. Further, many small molecule drugs are hydrophobic in nature, so soluble delivery into an aqueous environment presents a challenge. As such, one biomaterial approach to antigen‐specific tolerance is to exploit the immunomodulatory potential of small molecule drugs.

### 2.1 Materials Shift mTOR Inhibitors from Suppressive to Tolerizing Drugs

The mechanistic target of rapamycin (mTOR) has been one of the most targeted pathways over the past decade to induce immune tolerance using biomaterials. mTOR is a central cell metabolic pathway that regulates the manner in which cells respond to changing nutrient availability and consumption. In immune cells, inhibitors of mTOR, such as rapamycin and more recent analogs, are commonly used immunomodulators and immunosuppressives. mTOR inhibitors skew T cell responses away from effector and inflammatory subtypes toward regulatory and memory phenotypes and affect dendritic cell maturation and tolerance.^[^ ^28^, ^29^, ^30^, ^31^ ^]^ Delivered systemically, mTOR inhibitors broadly suppress immune responses, which has made these drugs components of the life‐long management transplant recipients undergo to minimize or slow rejection.^[^ ^32^, ^33^ ^]^ In models of autoimmunity, delivering these drugs in a material modified the effect of mTOR inhibitors from broad suppression to antigen‐specific tolerance. In some cases, delivery of mTOR inhibitors alone using material strategies has been used to treat autoimmunity and autoinflammation without broad suppression. While mTOR as a target for tolerance using biomaterials has been thoroughly studied,^[^ ^34^, ^35^ ^]^ this section will focus primarily on recent developments.

**Figure 2:**
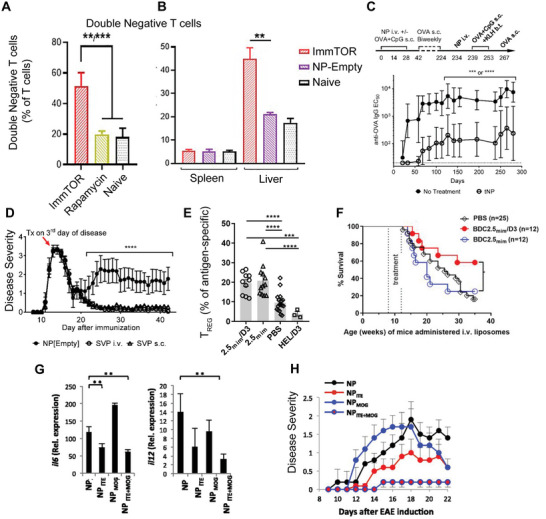
Small molecule approaches for tolerance. A) Double negative T cells expression following rapamycin NP (ImmTOR) treatment in livers and compared to B) spleens. A,B) Reproduced under the terms of the Creative Commons Attribution 4.0 International License.^[^ ^36^ ^]^ Copyright 2021, The Authors. Published by Frontiers Media S.A. C) Anti‐OVA titers in response to OVA sensitization and treatment with OVA rapamycin NPs with repeated rechallenge. D) Disease severity (EAE score) among mice treated with control (empty) or antigen‐rapamycin NPs administered via multiple routes. C,D) Adapted with permission.^[^ ^40^ ^]^ Copyright 2015, National Academy of Sciences. E) Antigen‐specific T_REG_ after antigen (2.5 mim)/calcitriol (D3) treatment compared to control. F) Diabetes‐free survival after treatment with antigen/calcitriol (red), control (black), or antigen (blue) particles. E,F) adapted with permission.^[^ ^48^ ^]^ Copyright 2020, American Association of Immunologists. G) Inflammatory gene expression among groups treated with antigen (MOG) and ITE constructs and H) disease severity after treatment. Mice were treated IP weekly starting on EAE day 0. G,H)Adapted with permission.^[^ ^57^ ^]^ Copyright 2012, National Academy of Sciences.

As alluded to, recent work has highlighted the ability of biomaterials to enhance the tolerogenicity of mTOR inhibitors through encapsulation and codelivery with antigen. In a recent work, rapamycin was encapsulated in nanoparticles (NPs) and delivered intravenously with the goal of generating immune tolerance.^[^ ^36^ ^]^ NPs enabled preferential uptake by liver APCs and altered T cell phenotypes in the liver. NP treatment ultimately protected against an inducible model of inflammation. Interestingly, the proportion of double negative T cells (T cells that express neither CD4 nor CD8) was found to significantly increase in the liver following treatment but not in other tissues (**Figure** **2**A,B). Double negative T cells have been documented to have a tolerizing role in immune responses,^[^ ^37^ ^]^ though the mechanism of the development of this population in this study is not clear. An additional possibility is that these could be invariant natural killer T lymphocytes (iNKT cells), which have recently been demonstrated to be reprogrammable for tolerance.^[^ ^38^ ^]^ This work highlights how a suppressive drug—rapamycin—can be focused to a less broadly suppressive effect through particle delivery, as these effects were localized to where particles were distributed and taken up. While this approach was protective against autoinflammation, the claim of tolerance would be more supported with delivery of antigen and analysis of antigen‐specific populations. A similar approach has been extended to specifically prolonging transplant survival by another group using subcutaneous rapamycin particle delivery^[^ ^39^ ^]^ which enabled uptake by relevant APC populations. While not autoimmunity, this serves as another example of how localization to a specific cell population using particles can prolong graft survival while modifying responses to graft antigens without broad suppression. However, a key limitation of these approaches is that without codelivering antigen, there is less control over which regulatory populations are expanded, requiring relevant antigen to be present in the system already, such as a graft.

To increase control over the specificity of tolerance, many groups have developed systems that codeliver antigen and mTOR inhibitor. In an early example of this, polymer NPs loaded with antigen and rapamycin were able to drive tolerance in model antigens, hypersensitivity, and relapsing‐remitting experimental autoimmune encephalomyelitis (RR‐EAE)—a mouse model of MS that exhibits relapses and remissions rather than the progressive disease of EAE.^[^ ^40^ ^]^ Using the model antigen OVA, this group demonstrated that following treatment with OVA‐rapamycin NPs expanded antigen specific OT‐II T_REG_ cells and that B cell responses were restrained against rechallenge for over 200 days after cessation of treatment (Figure 2C). The long duration of effect after treatment highlights the durability of this approach. Further, in a model of MS, treatment with antigen‐rapamycin particles after onset of disease protected against relapse when administered therapeutically (Figure 2D) demonstrating an ability to potentially reverse ongoing inflammation. Additional groups have coencapsulated other self‐antigens and rapamycin to promote protection in the context of the autoimmune diseases rheumatoid arthritis, type 1 Diabetes (T1D), and vitiligo.^[^ ^41^, ^42^, ^43^, ^44^ ^]^ Taken as a whole, biomaterial encapsulation of drugs alone or with self‐antigen enables tolerance induction via a traditionally suppressive drug class—mTOR inhibitors—highlighting the utility of materials in this application.

### 2.2 Encapsulation Enhances Efficacy of Metabolic Cues for Tolerance

While mTOR inhibitors are one of the most widely used classes of immunomodulator, a number of biomaterials have focused on other metabolic modulators and metabolites for tolerance. One example is the delivery of vitamin A and D derivatives. The vitamin A derivative all‐trans retinoic acid is a signaling molecule that contributes to immune tolerance to antigens in the gut microbiome.^[^ ^45^, ^46^ ^]^ In particular, retinoic acid can promote the differentiation of tolerizing APC populations and regulatory T cells via retinoid receptors—in some cases stabilizing these populations in inflammatory contexts. Leveraging this ability, multiple groups have used coencapsulation of antigen and retinoic acid in liposomes and polymer particles to expand regulatory T cells—a population important for specific suppression of autoimmunity—both in vitro and in vivo contexts.^[^ ^42^, ^47^ ^]^ The goal of these studies is to use delivered antigen to expand antigen‐specific cells, while retinoic acid promotes differentiation of those expanded cells into regulatory cells.

In addition to vitamin A derivatives, the vitamin D derivative calcitriol has also been used in biomaterials to enhance antigen‐specific outcomes. calcitriol inhibits NF‐*κβ*, a critical transcription factor for activation of immune cells such as APCs. The rationale for codelivery with antigen is that the inhibitor will prevent inflammatory responses in APCs presenting antigen, thus allowing for antigen presentation in a noninflammatory or regulatory context. One group showed that delivery of calcitriol with a T1D peptide antigen in a liposome promoted the expansion of antigen‐specific regulatory T cells as measured by tetramer^[^ ^48^ ^]^(Figure 2E). Interestingly, despite increases in T_REG_ from an antigen liposome without calcitriol, efficacy against disease required both components (Figure 2F). The authors showed that a population of Interleukin (IL)‐10 producing non‐T_REG_ antigen‐specific population was induced from calcitriol/antigen liposomes. The IL‐10 producing population was hypothesized to be the driving force in protection against disease. In a follow‐up study, the authors tested whether delivery of a CD8 epitope would be similarly efficacious, as CD8 cells are responsible for the destruction of islets in T1D.^[^ ^49^ ^]^ While delivery of the calcitriol and CD8 epitope delayed development of disease, delivering CD4 epitope formulations with the CD8 epitope formulations caused a loss of efficacy rather than an additive or synergistic effect. While the cause of this loss of efficacy is not clear, it is possible that targeting CD4 cells and CD8 cells simultaneously acts in a competitive manner. This highlights the need for careful antigen selection and that more is not always better. Calcitriol has also been codelivered with model antigens to promote tolerizing outcomes as well.^[^ ^50^, ^51^ ^]^

Beyond delivery of metabolites, small molecules that inhibit or otherwise interact with metabolic pathways in immune cells have also emerged as strategies to combat autoimmunity using biomaterials. One example of this is modulation of glutamate metabolism. APCs such as dendritic cells alter their cytokine secretion profile as a function of changes in glutamate metabolism, thus directly impacting differentiation of activated T cells.^[^ ^52^ ^]^ The small molecule N‐Phenyl‐7‐(hydroxyimino) cyclopropa[b]chromen‐1a‐carboxamide (PHCCC) is an allosteric enhancer of the glutamate receptor on dendritic cells and promotes a shift toward tolerizing cytokine productions. However, PHCCC is poorly soluble and has toxicity issues, making systemic delivery difficult. Our lab has demonstrated that PHCCC can be effectively loaded into liposomes that provide controlled release of drug over time, mitigating toxicity and solubility issues for this compound.^[^ ^53^, ^54^ ^]^ Further, PHCCC liposomes delayed disease onset in a mouse model of MS—EAE, while reducing proliferation of autoreactive T cells. However, as protection was not sustained and no antigen was delivered, this result may have been the result of a transient suppression, rather than antigen‐specific tolerance. Additionally, this approach could be enhanced by codelivering antigen to potentially expand T_REG_ populations under noninflammatory conditions.

Another metabolic regulator, the aryl hydrocarbon receptor, has been targeted by biomaterial approaches for tolerance. The aryl hydrocarbon receptor is a sensor that responds to changes in a cell's environment such as oxygenation. In immune cells such as dendritic cells, activation of this pathway can promote tolerizing phenotypes over inflammatory ones that cause downstream T cell differentiation toward T_REG_.^[^ ^55^ ^]^ Recent work has taken advantage of this tolerizing effect by coencapsulating 2‐(1′H‐indole‐3′‐carbonyl)‐thiazole‐4‐carboxylic acid methyl ester (ITE), an aryl hydrocarbon receptor agonist, and MS self‐antigen peptide. By coencapsulating these components, self‐antigen is more likely to be presented by tolerized dendritic cells, enhancing antigen‐specific T_REG_ expansion.^[^ ^56^, ^57^ ^]^ Tolerizing DC development was underscored by reducing the expression of the inflammatory cytokines IL6 and IL12 in DCs (Figure 2G). Additionally, treatment with NPs containing both ITE and antigen was more effective at reducing EAE disease severity in mice (Figure 2H). In follow‐up work, the same group demonstrated efficacy across several EAE disease models—demonstrating the robustness of this approach. The examples in this section highlight how immune metabolic modulation either through metabolite delivery or small molecule drug delivery can be enhanced by the advantages offered by biomaterials. Future work in this area could expand to include material delivery other classes of immune metabolites and metabolic modulators for with regulatory capacities such as short‐chain fatty acids and metabolites such as itaconate.

## 3 Biomaterials Enhance Tolerizing Effect of Biologic Drugs

While Section 2.2 discussed how biomaterials can manipulate immune pathways using small molecule drugs and metabolites for antigen‐specific tolerance, materials also enhance tolerance induction via higher molecular weight biologics and macromolecules—such as proteins and peptides. Similar to Section 2.2, materials allow for codelivery of antigen with tolerizing signals and cytokines to control expansion of antigen‐specific regulatory cell populations. In addition, biomaterials allow for control over how peptide and other macromolecule ligands are interacted with by target cells—for example, targeting cytokines to membrane receptors on the cell surface or delivering antigen to intracellular compartments for subsequent presentation. Further, biomaterial delivery allows for more targeted delivery of molecules such as cytokines that would otherwise have potentially toxic systemic effects and short circulation times. Increasing circulation times is potentially dependent on the type of biomaterial and administration route. For example, intravenous nanoscale materials may still be quickly cleared by scavenger cells, whereas controlled release microparticles (MPs) may allow sustained delivery of cargo at an injection site. This section will cover efforts to promote tolerance that use biologics and macromolecules—peptides and proteins, sugars, or nucleic acids—including some combination approaches that also include small molecules.

### 3.1 Materials Allow for Potent and Targeted Tolerance Induction Via Cytokines While Mitigating Off‐Target Effects

Inducing regulatory cell types and suppressing inflammatory cell types are some of the key levers that potential therapeutic approaches for tolerance seek to manipulate. Cytokines such as Transforming Growth Factor β (TGF‐*β*)and IL‐10 drive T_REG_ polarization and function, which makes these and related signals powerful tools tolerance induction.^[^ ^58^, ^59^ ^]^ However, therapeutic cytokine delivery has multiple challenges including potential toxicity and unfavorable side effects if delivered systemically as well as short half‐lives.^[^ ^60^ ^]^ TGF‐*β* is one of the principle regulatory cytokines, playing a role in T_REG_ induction and suppressing inflammatory responses in other T cell subtypes.^[^ ^61^, ^62^ ^]^ However, systemic delivery of TGF‐*β* has demonstrated to have dangerous off‐target effects such as lung fibrosis, making targeted approaches desirable.^[^ ^63^ ^]^ As such, many groups have exploited this role of TGF‐*β* through encapsulation and codelivery with antigen to treat and prevent autoimmunity in a variety of systems. In a recent study, PLGA MPs loaded with TGF‐*β* were used to prolong survival in islet allograft transplantation.^[^ ^64^ ^]^ In this model, pancreatic islets are chemically depleted from mice, then mismatched donor islets are grafted into hosts, serving as a model for transplant rejection. The authors implanted TGF‐*β* MPs alongside the islet transplants to set up a controlled release of tolerizing cytokine in the graft environment—thus avoiding systemic treatment with TGF‐*β* and focusing on T cells in the graft, a large portion of which would be expected to be graft specific. The result was development of T_REG_ specific for allograft antigens that were functionally suppressive ex vivo; however, despite functional T_REG_ generation, the grafted islets did not have prolonged survival in hosts, indicating the T_REG_ were either not protective or possibly too few. This result suggests antigen codelivery in some systems may be critical for sufficient expansion of protective T_REG_. While this example was in the context of islet transplant, it could also serve as a potential approach to treat islet autoimmunity in T1D.

**Figure 3:**
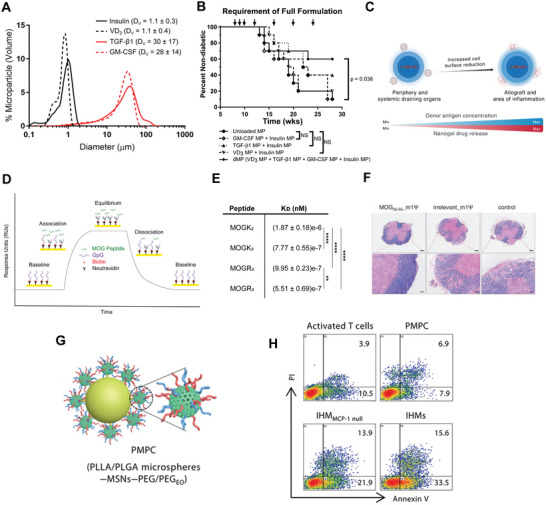
Biologic drugs used in biomaterials for tolerance. A) Particle sizing measurements of dual‐sized MP system. B) Diabetes‐free survival as a function of dual‐particle formula. A,B)_Reproduced with permission.^[^ ^68^ ^]^ Copyright 2019, American Chemical Society. C) nanogel “backpack” system that is T cell antigen‐signaling responsive. Reproduced with permission.^[^ ^71^ ^]^ Copyright 2020, AAAS.D) Schematic of surface plasmon resonance binding curve. E) K_D_ calculations from SPR as a function of different cationic amino acid modifications. D,E) reproduced with permission.^[^ ^76^ ^]^ Copyright 2021, American Chemical Society. F) Spinal cord H&E staining following treatment with MOG m1*ψ* mRNA therapy showing tissue damage in untreated mice. Reproduced with permission.^[^ ^82^ ^]^ Copyright 2021, AAAS. G) Schematic of multistage drug delivery for FasL MCP delivery. H) apoptotic cell numbers (Annexin V) following multistage drug delivery treatment. G,H) reproduced with permission.^[^ ^85^ ^]^ Copyright 2021, AAAS.

TGF‐*β* has also been delivered in conjunction with antigens and additional signals for tolerance. One approach being studied is a dual‐sized MP system that delivers a combination of antigens, small molecules, and cytokines to promote tolerogenic APCs for autoimmunity.^[^ ^64^, ^65^, ^66^, ^67^, ^68^, ^69^ ^]^ This approach takes advantage of the control of MP size to load intracellular cues (antigen, vitamin D3) into small MPs and extracellular cues (TGF‐*β*, granulocyte macrophage‐colony stimulating factor) into large MPs. The smaller particles are phagocytosed by target APCs, delivering cargo intracellularly, while the larger particles are too large for phagocytosis, instead releasing cargo into the extracellular space (**Figure** **3**A). This approach has demonstrated efficacy in producing APCs capable of inducing T_REG_ that are protective in models of T1D (Figure 3B), EAE, and rheumatoid arthritis. However, the antigen‐specificity of the expanded T_REG_ was not tested. The dual particle system highlights how the tunability of biomaterials can allow exquisite control over signal localization, including where in a cell a signal is encountered. While TGF‐*β* is critical for induction of T_REG_, IL‐10 is another critical cytokine for tolerance induction and suppression of inflammatory cells. While produced by many cell types, it is one of the ways in which T_REG_ and tolerizing APCs suppress inflammation. Leveraging this immunosuppressive activity, in a recent study, one group designed polymer NPs loaded with myelin antigen and IL‐10 to treat EAE.^[^ ^70^ ^]^ The team showed that subcutaneous treatment with these NPs reduced disease severity in mice while decreasing inflammatory cytokine production. Both antigen and IL‐10 were required for potent efficacy, but the claim of antigen‐specific tolerance would be strengthened by a control antigen as well as demonstration of unsuppressed healthy immunity after treatment. The described approaches highlight how suppressive cytokines generated by the immune system can be used for tolerance when delivered via materials as well as the modularity of material encapsulation for fine‐tuning desired immune responses.

In addition to the induction of antigen‐specific T_REG_, biomaterials delivering cytokines have been leveraged to enhance and sustain adoptively transferred antigen‐specific T_REG_. In this approach, T_REG_ are exogenously expanded then injected into recipients as a therapy for autoimmunity. In recent work, one group designed nanogels—NP scale hydrogels—that could be conjugated to T_REG_. These nanogels “backpacks” were loaded with IL‐2 and designed to be sensitive to local T cell receptor signaling^[^ ^71^ ^]^ (Figure 3C). The rationale for this approach is that T_REG_—once adoptively transferred—must be maintained to exert protection over time. T_REG_ express the high‐affinity IL‐2 receptor, CD25, and require IL‐2 signaling for maintenance.^[^ ^72^ ^]^ While systemic delivery of IL‐2 risks activating proinflammatory cell populations, release of IL‐2 in TCR‐rich signaling areas such as an allograft or a tissue experiencing autoimmunity can selectively sustain antigen‐specific populations. Use of these nanogels in conjunction with adoptively transferred T_REG_ significantly prolonged allograft survival in recipient animals. While this example was in the context of transplant tolerance, it could potentially be viable for autoimmune therapy as well. This approach demonstrates how specific biomaterial delivery can supplement antigen‐specific cellular therapies in addition to being used to induce therapeutic cells.

### 3.2 Materials Enable Blunting of Toll‐Like Receptor Pathways Via Nucleic Acid and Peptides

In addition to cytokine‐mediated pathways, another emerging set of pathways with roles in autoimmunity are toll‐like receptors (TLRs). TLRs are innate immune receptors that are expressed both intracellularly and extracellularly that recognized conserved pathogenic patterns such as bacterial flagella or viral double‐stranded RNA.^[^ ^73^, ^74^ ^]^ Signaling through these pathways activates immune cells and tunes downstream responses to align with the class of detected pathogen. While classically understood to mediate immunity to infection, recent studies have shown that TLR expression is also upregulated in cases of autoimmunity, underscoring their potential as targets for tolerance.^[^ ^75^ ^]^ In particular, TLR inhibitors have been studied as an approach to restrain autoimmunity. Many ligands and antagonists for TLR signaling are macromolecules such as nucleotides and peptides. Both peptides and nucleotides delivered in soluble form can be quickly degraded in the extracellular space by nucleases and proteases, making them potentially good candidates for material delivery in tolerance. Further, codelivery of antigen and TLR antagonist is ideal because it allows for antigen to be presented by APCs with TLR activation blocked, driving expansion of tolerizing cell populations. Importantly, depending on the TLR being targeted, expression is localized within a cell—for example, TLRs 3, 7, 8, 9, 11, 12, and 13 are on the endosome,^[^ ^73^ ^]^ so ligands for these receptors are most effective when phagocytosed. This control has been leveraged using biomaterials to change the context in which antigens are presented to promote tolerance in inflammatory setting. Our lab has developed a carrier‐free platform to codeliver TLR antagonist and myelin self‐antigen for EAE. The TLR9 antagonist GpG is a negatively charged nucleic acid that acts to inhibit endosomal TLR9. To form a carrier‐free material, the GpG is complexed with self‐antigen modified with cationic amino acid residues, thus allowing for self‐assembly of components either via layer‐by‐layer assembly or polyplexing—mixing both components together in solution.^[^ ^76^, ^77^, ^78^, ^79^, ^80^ ^]^ We have shown that these complexes can completely prevent disease in mice with EAE while still enabling mice to mount a successful immune response to a vaccine antigen. We also used surface plasmon resonance to test how modifying antigen charge affects the binding affinity of antigen to GpG and found arginine‐modified peptides had a smaller dissociation constant than lysine‐modified (Figure 3D,E), informing potential future self‐assembly strategies. This approach overcomes questions of innate immunogenicity of biomaterial carriers by delivering exclusively immunotherapy cargo. However, additional characterization of how charge modification and additional residues impact innate immunogenicity could be further explored. For example, altered charge could impact interaction with endogenous proteins and antidrug antibody production.

In addition to antagonizing TLR signaling, another approach to achieve tolerance is to evade TLR signaling altogether. mRNA vaccines have recently gained attention due to their role in vaccine development for the SARS‐CoV‐2 virus.^[^ ^81^ ^]^ These vaccines deliver mRNA encoding viral antigen in liposomes, which when taken up by cells induce expression of viral antigen. Simultaneously, the mRNA acts as a ligand for TLR7—a TLR that recognizes single‐stranded RNA. Signaling down this pathway increases the immune response to the antigen. In a similar approach, a noninflammatory mRNA vaccine was developed for EAE in mice.^[^ ^82^ ^]^ This approach is designed to deliver mRNA encoding self‐antigen to prevent and treat EAE. Expression of self‐antigen without inflammatory signaling could drive anergy and regulatory cell development rather than autoimmunity. To avoid TLR7 activation, the antigenic mRNA for this vaccine was modified with a pseudouridine group, allowing for expression of the self‐antigen without inflammation. mRNA is negatively charged, which makes delivery through the negatively charged cell membrane unfavorable. Additionally, as mentioned previously, free mRNA is subject to swift degradation by nucleases in the extracellular space. To overcome this, the authors used a liposome delivery system to encapsulate the mRNA, protecting against degradation and shielding negative cargo charge to facilitate uptake. Using this approach yielded strong protection against EAE while also driving increased induction of antigen‐specific T_REG_ as measured via FoxP3 expression. A more comprehensive characterization of T_REG_ could be obtained by staining for CD25 expression as well_._ Further, treatment protected against central nervous system destruction via demyelination (Figure 3F). This approach highlights how even a simple material such as a liposome and simple antigen modification can drive potent tolerizing outcomes.

### 3.3 Tolerance Can Be Induced Using T Cell Checkpoint Ligands

While much of the previous sections focused on tolerance induction via expansion of regulatory cell populations, treating autoimmunity can also be addressed by mechanisms that selectively reduce the number and functionality of inflammatory antigen‐specific cell populations. This can be accomplished through anergy and deletion of inflammatory cell populations. Approaches that seek anergy and deletion require precise targeting of antigen‐specific pathologic cells both for efficacy and safety—broad deletion of T cell populations could result compromising healthy immunity. Thus, material design parameters are ideal for targeting these mechanisms. Fas‐FasL signaling is one of the checkpoints on T cell activity, allowing the immune system to return to homeostasis after fighting infection.^[^ ^83^, ^84^ ^]^ T cells that sense FasL via Fas reduce inflammatory activity and undergo programmed cell death. The goal of using FasL for autoimmune prevention and therapy is to target FasL to self‐reactive T cells and program them for deletion to restore tolerance without compromising healthy immunity. Leveraging FasL signaling and Monocyte Chemoattractant Protein 1 (MCP‐1), one group developed a multistage drug delivery system (Figure 3G) that recruited and suppressed antigen‐specific cells in the multiple models of autoimmunity.^[^ ^85^ ^]^ The design rationale of these particles was to have an initial release of MCP‐1, which specifically attracts activated T cells—during active autoimmune inflammation, the authors hypothesized most activated T cells are pathologic. Once recruited via MCP‐1, T cells encounter conjugated FasL, inducing apoptosis activated cells. The authors showed that these particles did increase the proportion of apoptotic cells as measured by Annexin V (Figure 3H) and reduced disease score in a mouse colitis model. Further, these particles, when also encapsulating self‐antigen were able to protect against EAE in a specific manner compared to control antigen, which was unable to drive protection. The requirement of self‐antigen for efficacy supports this as a potentially selective approach. However, an approach such as this could potentially eliminate activated T cells fighting infection or cancer, as the MCP‐1 and FasL mechanism do not distinguish based on antigen‐specificity. This approach demonstrates that biomaterials can allow for tolerance via methods without necessitating direct targeting of regulatory cells, but instead promoting apoptosis of inflammatory cells. A potential limitation of this approach is whether this would protect against a relapse of autoimmunity—such as in relapsing‐remitting MS—without the induction of a maintained regulatory cell population. Deletion of autoreactive cells may only provide transient benefit in a case where there is a recurring source of autoinflammation.

### 3.4 Inhibitory Cell Surface Receptors Can Be Targeted for Tolerance Using Materials

**Figure 4:**
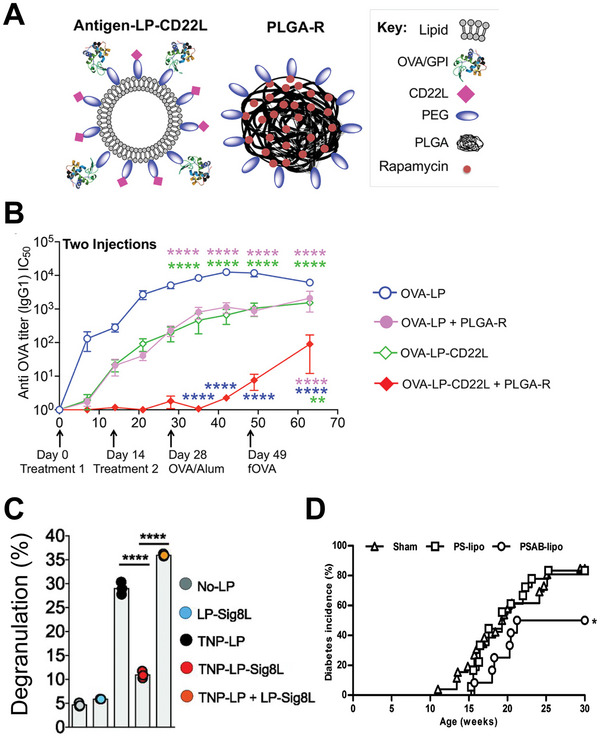
Additional biologics used in biomaterials for tolerance. A) Schematic of dual CD22L and rapamycin particle system. B) Anti‐OVA antibody production after treatment with one or both particle formulae. A,B) reproduced with permission.^[^ ^88^ ^]^ Copyright 2021, American Chemical Society. C) Proportion of cells degranulated (as measured by *β*‐hexaminadase release) as a function of antigen (TNP) or Siglec 8 (sig8L) loaded particles. Adapted with permission.^[^ ^89^ ^]^ Copyright 2021, American Association of Immunologists. D) Prevention of T1D using phosphotidyl serine liposomes loaded with antigen. Reproduced under the terms of the Creative Commons Attribution 4.0 International License.^[^ ^91^ ^]^ Copyright 2015, The Authors. Published by PLOS.

Cell surface markers that promote tolerance and suppress reactivity in immune cells besides T cells and APCs have also been effectively used to drive tolerizing outcomes. Sialic acid binding Ig‐like lectin (Siglec) receptor signaling plays a range of roles in immune cell interaction and modulation; some of these functions are inhibitory or regulatory in nature.^[^ ^86^ ^]^ CD22 is a Siglec expressed on B cells that, when activated, reduces antibody production in targeted B cells as well as apoptosis. Targeting CD22 ligands to autoreactive B cells in autoimmunity has the potential to reduce production of autoantibodies that contribute to disease pathology. CD22 suppressive function on B cells requires CD22 receptors to be spatially close to B cell receptors within the cell membrane. Materials allow codelivery of CD22L tethered to antigen to target CD22 and autoreactive B cell receptors simultaneously while bringing them closer together. Multiple groups have used antigen and CD22L delivery to reduce production of antibodies in the context of model antigens, arthritis, and hemophilia.^[^ ^87^, ^88^ ^]^ This approach has also been demonstrated to synergize with other tolerizing approaches such as rapamycin NPs to further reduce antibody production (**Figure** **4**A,B). As discussed in other examples the claim of antigen‐specific tolerance could be enhanced through additional controls such as an irrelevant antigen group. CD22L delivery highlights how B cells can potentially also be cellular targets for tolerance. Targeting Siglec‐family surface receptors has potential relevance in suppressing other cell types that cause autoimmunity and allergy. Siglec‐8 is an inhibitory receptor present on allergy‐driving eosinophils and mast cells. Instead of reducing antibody production, Siglec‐8 inhibition in these cells inhibits degranulation of histamines and other mediators of allergy. Recent work used this pathway to codeliver antigen and Siglec‐8 agonist to treat a mouse model of allergy.^[^ ^89^ ^]^ Mice treated with Siglec‐8 and antigen displaying NPs were protected in a model of anaphylaxis, while treated mast cells were desensitized against rechallenge with antigen. Further, degranulation was only prevented when antigen and Siglec‐8 were displayed on the same liposome (Figure 4C). In both examples, the location of Siglec ligand and antigen was critical. Surface presentation of components was necessary to pull Siglec receptors close to antigen recognition complexes. If antigen and Siglec ligand were delivered via encapsulation without joined antigen and Siglec ligand, these approaches may not be viable. Taken together, these approaches highlight how other cell populations can be valuable for inducing tolerance. Further, the biology of the targeted pathway is critical for informing the material delivery method.

Beyond regulatory ligands used to control cell types of interest, several groups have used the anti‐inflammatory effect of apoptotic cells to promote tolerance to antigens. Apoptotic cell death is tightly regulated and commonly occurs as a part of healthy tissue function, thus immune cells recognize apoptotic cells in a noninflammatory manner.^[^ ^90^ ^]^ This contrasts with necrotic cells, which occur in cell or tissue damage, situations that promote inflammatory responses. The noninflammatory response to apoptotic cells can be used to promote tolerance to antigens in autoimmunity. When cells become apoptotic, they have increased expression of phosphotidyl serine on the outer leaflet of their cell membrane—a residue usually on the inner leaflet on living cells. This residue is recognized by antigen presenting cells and promotes a tolerizing response rather than an inflammatory one. Recently, several groups have used liposomes loaded with antigen and phosphotidyl serine to promote tolerance in T1D.^[^ ^91^, ^92^ ^]^ The rationale for this approach is to mimic an apoptotic cell with a liposome displaying phosphotidyl serine and encapsulating T1D relevant antigen. APCs encounter these mimic apoptotic cells and take them up, adopting a regulatory APC phenotype. The authors demonstrate resultant antigen‐specific T cell expansion but do not demonstrate that these are a regulatory cell population. The authors found that these liposomes protected mice against diabetes onset as compared to a control group, though the effect was modest (Figure 4D). It is possible this approach could be strengthened through addition of other regulatory cues. Similar to Siglec, above, the crux of this approach is effective materials selection. Use of a liposome with incorporated phophotidyl serine mimics an apoptotic cell membrane. Without liposomal delivery, it would be challenging to engage this tolerizing mechanism as effectively. Additional cell‐mimicking approaches that display antigens and inhibitory ligands could build on this approach.

## 4 Modifying Antigen Material Properties and Immunogenicity Can Drive Tolerance

Previous sections discussed small molecule and biologic signals that have been used to induce tolerance using biomaterials. These approaches allow for more precise delivery and control over tolerizing pathway activation in coordination with antigen delivery. However, synthesis and delivery of these biomaterials requires combination of multiple components within a carrier, wherein a large proportion of the delivered therapeutic is carrier rather than cargo. Further, many peptide antigens have low immunogenicity by themselves, motivating strategies that increase antigen immunogenicity. To address these issues, recent work has focused on modifying the material properties of antigens or fusing them with other signals. This concept leverages material properties such as surface charge and molecular weight to alter biodistribution and interaction with immune cells to improve regulatory outcomes.

### 4.1 Carbohydrate Modification of Antigens Focuses Signals in Tissues and Enhances Tolerance Induction

**Figure 5:**
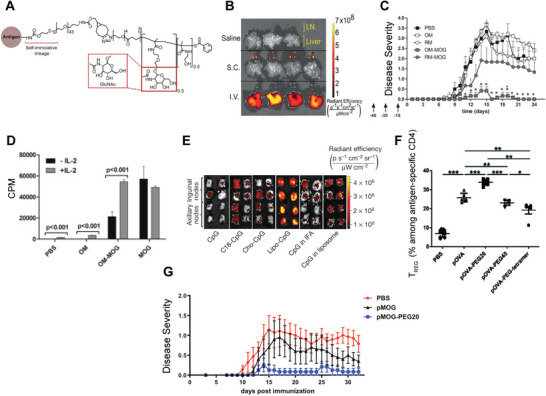
Materially modified antigens for tolerance. A) Schematic of GluNaC modified antigens with self‐immolating linkage. B) IVIS depicting increased accumulation in livers via I.V. treatment. A,B) Reproduced under the terms of the Creative Commons Attribution 4.0 International License.^[^ ^94^ ^]^ Copyright 2021, The Authors. Published by Frontiers Media S.A. C) Paralysis level (disease severity) of mice treated with mannosylated antigens and D) proliferation in response to antigen with and without IL‐2 to measure anergy. C,D) reproduced with permission.^[^ ^96^ ^]^ Copyright 2015, Elsevier. E) Biodistribution in axillary LNs via IVS of lipo‐modified immune cargo (CpG). Reproduced with permission.^[^ ^98^ ^]^ Copyright 2014, Springer Nature. F) Treg induction after treatment with PEGylated OVA and G) paralysis level (disease severity) after treatment with PEGylated disease antigen (MOG). Mice were treated 7 days before EAE induction. F) Reproduced under the terms of the Creative Commons Attribution 4.0 International License.^[^ ^100^ ^]^ Copyright 2020, the authors. Published by Frontiers Media S.A. G) Adapted with permission.^[^ ^101^ ^]^ Copyright 2021. SAGE Journals.

As discussed earlier in this review, one of the goals of materials‐based approaches is increased control over delivery to particular immune cell types and tissues. Conjugating carbohydrate groups (such as via glycosylation and mannosylation) to antigens is a strategy that has been used to increase uptake by particular subsets of APCs and increase trafficking to critical immune tissues such as lymph nodes while avoiding rapid clearance. Glycosylation both increases the material properties of molecular weight of delivered antigens and modifies the overall charge, favorably altering trafficking dynamics and uptake by cells, all accomplished without encapsulation within a material. Further, glycosylated antigens are preferentially taken up by innately tolerogenic APCs housed in the liver; thus, glycosylation is a potential approach for increased immunogenicity and tolerance. In recent work, peptide antigens were glycosylated to increase uptake by tolerizing hepatic APCs.^[^ ^93^, ^94^, ^95^ ^]^ By modifying antigens with N‐acetylgalactosamine or N‐acetylglucosamine and delivering intravenously, the authors were able to preferentially target the tolerizing hepatic APCs in the liver, allowing for increased generation of T_REG_ and suppression of autoimmune diabetes. Carbohydrate linkers are designed to be cleaved intracellularly, allowing for presentation of unmodified antigen by APCs after uptake (**Figure** **5**A). In a separate study, the authors demonstrated that with subcutaneous administration instead of intravenous, the modified antigens could be targeted to draining lymph nodes (Figure 5B) instead of the liver to induce tolerance via antigen‐specific CD4 and CD8 T cell responses. In a similar approach, another group modified myelin antigens for EAE with mannose fused to tumor mucin antigen to increase APC targeting via mannose receptors that are naturally expressed on APC populations.^[^ ^96^, ^97^ ^]^ Interestingly, while delivery of these antigens protected mice from developing EAE (Figure 5C), protection was not through the induction of T_REG_ but instead anergy of T_H_1 and T_H_17 cells that underpinned tolerance (Figure 5D). Anergy occurs when antigen is presented to T cells without additional cues and costimulation, and the resulting cells are not responsive to subsequent antigen stimulation. In exciting recent work, the authors demonstrated that this treatment was protective and therapeutic in humanized mice, underlining the translational potential of such approaches. Glycosylated and mannosylated antigens highlight how using material properties to modify simple soluble antigens can have potent tolerizing effects. Due to the multivalency of clinical autoimmunity, translation of approaches such as these may require delivery of multiple antigens dependent on which autoantigens are most prominent in a particular patient.

### 4.2 Modification with Amphiphilic Residues Allows Self‐Antigen Targeting to Lymph Nodes

One challenge facing the delivery of immunotherapies is achieving sufficient signal density in target tissues to produce a desired effect. Specifically, delivery to lymph nodes is important for therapies designed to impact T and B lymphocyte interactions; however, traditional routes of delivery such as subcutaneous result in a large proportion of the delivered cargo remaining at the injection site, and thus “invisible” to adaptive immunity. One of the strategies developed to increase lymph node targeting of cargo is leveraging the natural carrier protein albumin. One approach is to use modified immunogenic cargo with lipophilic moieties to generate amphiphilic antigens and modulators.^[^ ^98^ ^]^ These lipo‐modified antigens self‐assemble with albumin via hydrophobic interactions to “hitchhike” to lymph nodes via natural albumin trafficking. This approach has been shown to increase cargo delivery to lymph nodes (Figure 5E). More recently, amphiphilic antigen modification was used to protect against mouse T1D.^[^ ^99^ ^]^ The authors modified insulin B9‐23 with lipophilic residues and demonstrated increased lymph node draining and association with APCs. Further, they demonstrated efficacy against spontaneous diabetes development in non‐obese diabetic (NOD) mice. Though, it should be noted that efficacy was modest compared to delivery of soluble B9‐23 peptide. Taken as a whole, this work highlights how an approach developed to promote an inflammatory response toward a cancer can be modified to be advantageous at promoting stronger tolerogenic outcomes.

### 4.3 PEGylation of Antigens Increases Circulation and Protects Against Autoimmunity

In addition to glycosylation and amphiphilic modification, peptide circulation, and potency has been increased using PEGylation of antigenic peptides. The advantage of this approach is that PEGylation is a well‐characterized and widely used approach for increasing circulation time and reducing clearance of biologics. In a pair of manuscripts, PEGylated antigens were used to promote antigen‐specific tolerance and reduce inflammatory responses.^[^ ^100^, ^101^ ^]^ In a first publication, the authors demonstrated PEGylated OVA had increased the ability of OVA to induce antigen‐specific T_REG_ expansion (Figure 5F). Interestingly, this was dependent on the molecular weight of PEG used, underscoring the powerful impact of material selection on immunological outcomes in tolerance. It should be noted that the authors assessed T_REG_ using FoxP3, though additional costaining with CD25 would provide more complete characterization. In a subsequent publication on this work, the authors tested if this approach could reduce the severity of disease in an animal model. They tested delivery of PEG‐modified myelin antigen in the context of EAE and showed that this modified antigen could reduce EAE clinical score when delivered prophylactically (Figure 5G). However, this was not efficacious when delivered after the induction of EAE. This approach is encouraging, because it demonstrates that even simple material modifications can promote outcomes in a disease context; however, the limitations of efficacy show that a clinical approach may require some additional component to drive protection. Additionally, as the authors point out, PEGylation of drugs in the clinic is often hampered by the development of anti‐PEG antibodies that reduce efficacy which could be a concern moving this approach into the clinic.^[^ ^102^ ^]^

### 4.4 Modifying Antigen Immunogenicity Can Induce Tolerance

In addition to modifying antigens with functional groups to increase molecular weight or alter intracellular processing, the immunogenicity of antigens has been modified via fusion of multiple antigenic peptides or modification with ligands to promote more effective tolerance induction. In the context of T1D, one group developed a fusion antigen comprised of multiple T1D antigens (insulin, chromogranin A) and tested the functionality of this modified antigen in mouse models of both islet transplant and diabetes when delivered in a NP.^[^ ^103^, ^104^ ^]^ The authors tested the ability of hybrid peptide NPs to prevent onset of induced T1D. The authors showed these NPs protect against disease and increase the proportion of antigen‐specific T_REG_ in treated mice. In more recent work, the authors showed that these same NPs were also efficacious at increasing the survival of syngeneic islet grafts in diabetic mice. A related approach is the use of altered peptide ligands. Rather than fuse antigens together to increase antigenicity, altered peptide ligands change the amino acid sequence of an antigen to drive tolerance more favorably. In a recent study, the T1D autoantigen GAD was modified and delivered in a liposome to drive tolerance in T1D.^[^ ^105^ ^]^ To overcome the lower immunogenicity of delivering just a peptide antigen, the authors conjugated the antigen to a TLR2 agonist to increase uptake and presentation of the altered peptide. Delivery of this nanoparticulate system protected against T1D in mice and reduced inflammatory cytokine production among pathogenic cell populations. However, caution should be used particularly with altered peptide ligands in autoimmunity, as in some cases, they have exacerbated rather than alleviated disease.^[^ ^106^ ^]^ Another approach to increase the tolerizing effect of peptide antigens is to conjugate them to a tolerizing signal. As discussed in a previous section, Siglec receptor targeting on immune cells acts to decrease activity and inflammation across populations including B cells, dendritic cells, and allergenic cells. In a recent study, one group conjugated sialic acid to antigens to drive Siglec signaling during antigen recognition.^[^ ^107^ ^]^ The authors found these modified antigens effectively generated FoxP3+ antigen‐specific T_REG_ from naïve precursors. As in other examples, the characterization of T_REG_ could be strengthened through addition of CD25 costaining. This demonstrates how conjugating self‐antigens to tolerizing signals can increase the potency of antigenic therapies.

## 5 Biomaterials Localize Cargo in Tissues That Support Tolerogenic Responses

Although previous sections focused on mechanisms through which biomaterials enable drugs, signals, and modified peptides to induce tolerance, the context in which these cues are processed by cells can play an important role in eliciting a tolerogenic response. While some treatments rely on systemic delivery of cues, many strategies rely on harnessing cells and tissues that already support naturally tolerogenic environments. Particularly, engineered properties such as size, surface ligands, and chemical structure are of paramount importance to allow for improved biomaterial trafficking to or retention in these tissues. Improved tissue localization in combination with previously mentioned features such as codelivery, controlled release, and cargo protection are important for delivering cues in a precise manner to orchestrate desired tolerizing immune responses. This section will address how biomaterials localize their cargo in specific tissue types for engineering antigen‐specific tolerance.

### 5.1 Materials Allow Direct Re‐Engineering of the Lymph Node Microenvironment For Tolerance

**Figure 6:**
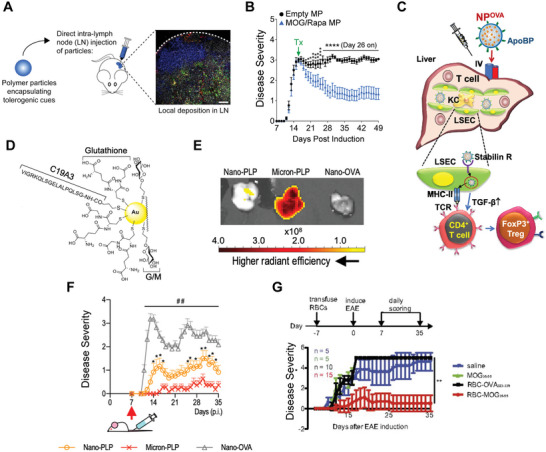
Tolerance induction through targeted tissue delivery. A) Schematic of direct LN injection. B) Disease severity (paralysis) of mice treated intra‐LN with MOG‐Rapa or control MPs at peak disease. A,B) Reproduced with permission.^[^ ^110^ ^]^ Copyright 2016, Elsevier. C) Schematic of strategy for liver targeting using Apolipoprotein B to generate T_REG_. Reproduced with permission.^[^ ^115^ ^]^ Copyright 2021, American Chemical Society. D) Gold NPs were conjugated with T1D antigen. Reproduced with permission.^[^ ^122^ ^]^ Copyright 2019, Elsevier. E) IVIS plots showing MP but not NP accumulation in lungs following IV injection. F) Disease severity of mice in a mouse model of MS treated intravenously with either MP or NP formulations. E,F) Reproduced with permission.^[^ ^127^ ^]^ Copyright 2020, AAAS.  G) Red blood cells (RBCs) modified with MOG antigens reduced severity of a model of MS in an antigen dependent manner. Reproduced with permission.^[^ ^128^ ^]^ Copyright 2017, National Academy of Sciences.

Biomaterials are powerful tools for colocalizing and retaining signals in the lymph node for antigen specific tolerance. As previously discussed, lymph nodes serve as specialized secondary lymphoid structures that orchestrate interactions between T, B, and dendritic cells to mount immune responses. Thus, localized intra‐lymph node (iLN) treatments have the potential to elicit a strong systemic immune response without the off‐target disadvantages of systemic therapies. However, soluble or nanoencapsulated immune signals are often too quickly cleared out of the lymph node after injection to deliver sustained cargo concentrations required for effective tolerance induction. To overcome clearance of soluble cues and poor drainage of traditional delivery routes, our lab developed an approach of direct lymph node injection of MP depots (**Figure** **6**A), specifically designed too large to drain from lymph nodes. Instead, the particles deliver a controlled release of immunotherapy cargo directly into the highly immunogenic lymph node microenvironment. We found that 2–3 µm sized PLGA MPs are diffusion‐limited based on their size and were retained in the lymph node much longer than nanoscale particles.^[^ ^108^ ^]^ Further, alterations to the lymph node microenvironment were highly localized to the injected lymph node.^[^ ^109^ ^]^ When loaded with myelin self‐antigen and rapamycin, iLN injected particles were shown to induce significant expansion of T_REG_ compared to soluble controls. Additionally, iLN MPs were able to reverse paralysis in mouse EAE in an antigen specific manner with a one‐time treatment at peak disease^[^ ^110^ ^]^(Figure 6B). Critically, delivery of the same MP formulae via traditional delivery routes (intramuscular) was ineffective at preventing paralysis, highlighting the need for tissue‐specific delivery. Additionally, treatment with a nondisease antigen particle was ineffective at preventing disease, underscoring the selectivity of this approach. Finally, biomaterial enabled retention of signals in the lymph node limits the potential for off target exposure of immunosuppressive agents, which would drastically improve upon currently available treatments that systemically suppress immune cells. Altogether, these studies highlight the necessity of biomaterials to localize and sustain cargo delivery in lymph nodes for engineering tolerogenic immune responses.

### 5.2 Liver APCs Naturally Tolerize Immune Cells and Are a Target for Biomaterial Localization

As alluded to in previous sections, APCs in the liver are a cell type predisposed to tolerance. Systemically delivered nanoscale particles can be designed for preferential uptake by naturally tolerogenic APCs through clatherin‐mediated endocytosis and may be used to induce antigen specific tolerance.^[^ ^111^ ^]^ In particular, liver sinusoidal endothelial cells (LSECs) have gained attention for their ability to attenuate disease through suppressive cytokine secretion, inhibitory marker upregulation, and T_REG_ cell expansion. It should be noted that LSECs are one of the primary scavenging mechanisms for nanoscale materials, so the precise diameter and targeting ligands may not be necessary for effective targeting for these approaches.^[^ ^112^ ^]^ In a model of bile duct inflammation using the model antigen OVA, iron oxide NPs ranging from 10 to 100 nm were shown to target LSECs, induce OVA antigen specific T_REG_ expansion, and inhibit OVA‐reactive CD8 T cell activity through antigen cross presentation.^[^ ^113^ ^]^ In a separate study, these authors achieved T_REG_ expansion through LSEC targeting NPs with self‐antigens in a mouse model of MS.^[^ ^114^ ^]^ Other researchers have used PLGA polymer to generate 200–300 nm particles that target LSECs based on particle size, and surface coated LSEC receptor targeting residues.^[^ ^115^ ^]^ This strategy relied of conjugating PLGA NPS with apolipoprotein B to target mannose receptors found extensively on LSECs (Figure 6C). It should be noted that the protein coronas typical on NPs often contain apolipoprotein, so conjugation of additional apolipoprotein may not be necessary for this effect.^[^ ^116^ ^]^ This led to increased production of the regulatory cytokine TGF‐*β* and induction of T_REG_ cells. Ultimately apolipoprotein B modified particles significantly reduced inflammation in an OVA model of allergy. Importantly, these studies highlight the ability of biomaterial delivery strategies that target the liver to attenuate both CD8 and CD4 driven autoimmune diseases.

### 5.3 Materials Enable Direct Targeting of Skin‐Resident Immune Cell Populations

The skin is an attractive target for antigen specific therapy due to the presence of tissue resident APCs that play an important role in the maintenance of peripheral tolerance to self‐antigens. Biomaterial delivery strategies have focused on localizing antigens in the skin to target these cells and leverage their ability to confer tolerance. One strategy relies on the use of microneedles: micrometerscale arrays that directly deposit cargo into the dermis. Microneedles are a highly modular platform that can be engineered to deliver cargo via surface coating, soluble release, or controlled degradation.^[^ ^117^, ^118^, ^119^, ^120^ ^]^ In one preliminary study looking at the uptake of pro‐insulin, coated stainless steel microneedle delivery was shown to elicit greater antigen‐specific activation of cells compared to intradermal injection due to the sustained release from the needles.^[^ ^121^ ^]^ In a separate study, insoluble T1D autoantigen was conjugated to naturally suppressive gold NPs (Figure 6D), and actively injected into the skin with microneedles.^[^ ^122^, ^123^ ^]^ In a highly clinically relevant model of ex vivo human skin, gold NPs with peptide were readily taken up by DCs and suppressed activation of antigen specific CD4 T cells in a dose‐dependent manner.^[^ ^122^ ^]^ Gold NPs have also been shown to increase systemic trafficking of insoluble T1D peptide to secondary lymphoid organs after injection into the skin, which may be useful for targeting multiple immune cell populations for a robust tolerogenic response.^[^ ^123^ ^]^ These studies highlight the wide range of materials that can be used to target delivery of autoantigens into the skin.

### 5.4 Materials Deliver Antigens to Take Advantage of Tolerogenic Cell–Cell Interactions in the Lung

Mucosal tissues such as the gut, sinus, and lungs are subject to innate tolerizing immunity due to interactions with innocuous foreign antigens. Among mucosal barriers, there is a hypothesis that the lungs serve an important role in autoimmunity as self‐reactive T cells traffic through the tissue before proceeding to inflamed tissues.^[^ ^124^, ^125^ ^]^ Recently, biomaterials have emerged as a method to target self‐antigen and immunotherapies to the lung to exploit these naturally tolerogenic T cell‐APC interactions. In one study, MS antigen conjugated to a hyaluronan backbone and delivered into the lungs reduced disease score and improved animal weight compared to unconjugated controls.^[^ ^126^ ^]^ In another study, the lungs were targeted via a combination of particle size control and injection route to treat a preclinical mouse model of MS.^[^ ^127^ ^]^ The authors found that PLGA MPs were preferentially retained in the lungs over NPs following intravenous injection (Figure 6E). MP formulae correspondingly provided stronger protection against a mouse model of MS (Figure 6F). These particles also yielded suppressive gene expression profiles for the lung, the central nervous system, and the mediastinal lymph nodes.

### 5.5 Leveraging Naturally Tolerogenic Apoptotic Cell Debris to Induce Tolerance

Apoptotic cell debris is trafficked for removal from the body by blood and contains many self‐antigens. Normally, T cells that recognize these self‐antigens without receiving activating signals become anergic. Biomaterial strategies have attempted to take advantage of this mechanism by conjugating autoimmune disease antigens to apoptotic cell debris ex vivo. Upon infusion, these conjugates will promote anergic responses form autoreactive T cells in circulation. Particularly, leveraging the clearance mechanisms of apoptotic erythrocytes is a possible mechanism to promote tolerance. This has been demonstrated for the MS peptide MOG_35‐55_ which was covalently linked to erythrocytes ex vivo.^[^ ^128^ ^]^ In a preclinical model of MS (EAE), preventative administration of these modified erythrocytes significantly lowered disease scores of mice in an antigen dependent manner (Figure 6G). Expanding on this, studies are now focused on conjugating antigens with erythrocyte binding scFv domains in vivo.^[^ ^129^ ^]^ Upon i.v. injection, these modified antigens covalently link to erythrocytes. This reduces the added complexity of ex vivo antigen‐erythrocyte conjugation and eliminates patient specific treatment requirements. The authors were able to show treatment with TER119‐p31 (diabetic p31 peptide linked to erythrocyte specific scFv) conferred protection to an aggressive model of diabetes in mice.^[^ ^129^ ^]^ Specifically, TER119‐p31 conjugates significantly improved uptake of the p31 peptide by splenic DCs and nonprofessional APCs and led to autoreactive T cell deletion. These results underscore the applicability of biomaterials for delivering antigens to selective physiological compartments to induce antigen specific tolerance.

## 6 Materials Can Be Used to Drive Tolerance Through Defined Antigen Display

While previous sections of this review discuss how biomaterials enable antigen‐specific tolerance induction through activation of tolerizing pathways or by modifying materials to target particularly tolerizing tissues, there has been a large body of research wherein delivery of antigen alone in a material alone can be used to promote tolerance. These approaches are largely based on display of antigens in noninflammatory contexts which can promote tolerance. This includes delivery of scaffolds that either allow antigen uptake in a controlled environment or divert inflammatory cells away from disease tissues. Another emerging approach is materials displaying peptide on MHC molecules to activate antigen‐specific T cells in the absence of costimulation to promote tolerance. Finally, this section will also cover particle delivery of antigens both conjugated to the particles and/or encapsulated within particles. Modification of material properties such as size and stiffness, as well as where in the particle antigen is located can be manipulated to drive tolerance over inflammation and allergy. Each of these approaches have their advantages and drawbacks, while underscoring how material properties can impact immunological outcomes.

### 6.1 Scaffolds and Soluble Antigen Arrays Allow Control Over Antigen Density and Location

**Figure 7:**
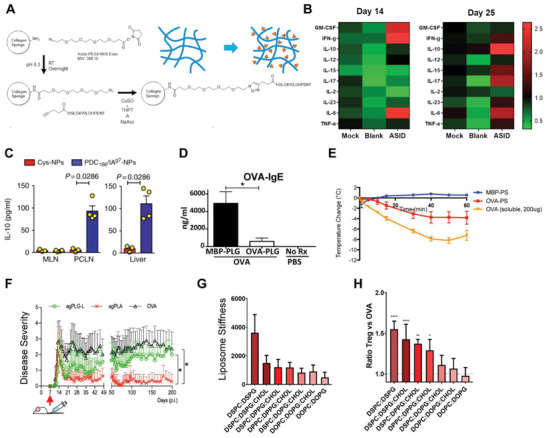
Biomaterial approaches encapsulating antigen alone. A) Schematic of collagen based immune decoy scaffold construction. B) Inflammatory gene expression among restimulated splenocytes among decoy (ASID) or control treated mice. A,B) Reproduced with permission.^[^ ^134^ ^]^ Copyright 2019, Elsevier. C) IL‐10 production among antigen stimulated B cells after treatment with pMHC (PDC/IA) constructs or control. Reproduced under the terms of the Creative Commons  CC‐BY license.^[^ ^138^ ^]^ Copyright 2019, The Authors. Published by Springer Nature. D) IgE production as a function of OVA‐PLG particle treatment or control. E) Temperature change (measure of anaphylaxis) as a result of treatment with conjugated or encapsulated OVA. D,E) reproduced with permission.^[^ ^146^ ^]^ Copyright 2016, National Academy of Sciences. F) Paralysis (disease severity) as a function of antigen loaded into distinct polymeric carriers. Reproduced with permission.^[^ ^151^ ^]^ Copyright 2019, Elsevier. G,H) Characterization of liposomes based on. G) AFM measured liposomal stiffness and H) Treg induction resulting. G,H) Reproduced with permission.^[^ ^158^ ^]^ Copyright 2020, Elsevier.

Encounter of antigen in the context of material scaffolds allows for control over the context in which these peptide antigens are interacted with while avoiding the issue of clearance for small peptide antigens. One group has developed scaffold‐like materials wherein self‐antigens are bound to a hyaluronic acid backbone—termed a soluble antigen array.^[^ ^130^, ^131^, ^132^, ^133^ ^]^ This material selection allows control over antigen density and molecular weight of the backbone—influencing T and B cell receptor engagement and biodistribution, respectively. Over several different published works, the authors demonstrate that these scaffolds can ameliorate autoimmunity in mice in the context of T1D and EAE when loaded with disease relevant self‐antigens. The proposed mechanism for these arrays is multifold. The arrays drain easily through lymphatics, increasing targeting of lymph nodes and the resident immune cell populations. Interaction with APCs in the absence of inflammation allows for presentation of antigen to T cells without co‐stimulation to reduce inflammatory responses. Additionally, repeating epitopes with varying valency can potentially better engage B cells compared to soluble antigens, while also demonstrating the unique ability of materials to test variables such as antigen density. In both the context of T1D and EAE in mice, this approach resulted in protection against disease and reduced severity of disease. In the context of T1D, the efficacy was dependent on the delivery of several epitopes, demonstrating that antigen selection, not just material delivery, may be critical for success in antigen‐specific therapies. The same group also developed an antigen‐specific immunological decoy in which self‐antigen was conjugated to a collagen scaffold^[^ ^134^ ^]^ (**Figure** **7**A). Rather than change the density of epitopes to alter immune signaling, the decoy was designed to divert pathologic antigen‐specific immune cells generated from EAE induction. Use of these decoys prevented the onset of EAE when implanted before disease onset, and the authors show recruitment of antigen‐specific cells to the scaffold and a reduction in inflammatory gene expression (Figure 7B). The proposed mechanism is that this exhausts the diverted cells, though this claim could be strengthened by assaying key T cell exhaustion markers such as PD‐1/CTLA4, which would be upregulated in activated and exhausted cell populations.

Another scaffold‐based approach uses a peptide‐based hydrogel for T1D. This material is a peptide scaffold that swells with water to form a gel. The authors had previously developed a peptide‐based hydrogel designed to act in inflammatory manner as a method to deliver cancer vaccines. They found the peptide hydrogel contained overlapping residues with several T1D antigens including insulin, proinsulin, and GAD.^[^ ^135^ ^]^ They hypothesized that these gels could potentially act as an antigen‐specific therapy for T1D. When administered to prediabetic mice, this hydrogel approach was able to reduce disease onset. While this is encouraging, the authors could have done more to demonstrate that the response occurring was antigen‐specific, such as by demonstrating successful vaccination against a foreign antigen in treated mice. Further, the authors demonstrated affinity for these gels for insulin receptor but did not compare to insulin as a control which would confirm that the affinity is physiologically relevant. They also did not confirm that residue that bound the insulin receptor was also the immunogenic portion of that epitope. Despite these potential drawbacks, this approach demonstrates how potentially powerful materials themselves can be in modulating disease. Hydrogel scaffolds composed of PLG polymer have also been used to prevent T1D through both the delivery of peptides in a noninflammatory contexts and as a way to deliver antigen‐specific T_REG_ for cellular therapies.^[^ ^136^, ^137^ ^]^ In both cases, material delivery in scaffolds enables control over the environment surrounding these cells and antigens.

### 6.2 Peptide‐MHC Constructs Promote Tolerance Via Antigen Presentation Without Costimulation

Another material that has been used to deliver antigen without explicit regulatory cues to promote tolerance are peptide‐MHC constructs on NPs. This material consists of an iron NP bound to a self‐antigenic peptide in an MHC molecule. The rationale of this approach is to mimic the context that self‐antigen would be encountered by T cells in vivo. Rather than self‐antigen being taken up and presented by APCs, T cells are able to interact with peptide‐MHC constructs directly, ensuring no costimulation is present during antigen presentation, avoiding inflammatory outcomes. This approach has demonstrated broad efficacy in treating a wide range of autoimmune disorders in mice, highlighting the modularity of the approach as well.^[^ ^138^, ^139^, ^140^, ^141^, ^142^ ^]^ The authors initially showed that peptide‐MHC class I NPs could promote regulatory and memory‐like CD8 cells through antigen stimulation in the absence of stimulus. Later work expanded this approach to peptide‐MHC class II constructs to target CD4 cells. Through these platforms, the authors have demonstrated tolerance in T1D and several liver autoimmune disorders in mice. Interestingly, these results are achieved through delivery of a single peptide antigen, without need for additional epitopes or explicit regulatory cues—instead using material to control how antigen is seen by T cells. Further, NP treatment resulted in the recruitment of regulatory B cells despite targeting CD4 cells (Figure 7C).

### 6.3 Conjugating Antigen to Particles Avoids Burst Release of Antigenic Cargo

Another approach used to delivery antigen in particulate form is to covalently bond self‐antigen to the surface of the particle or the matrix of the particle itself. The goal of these approaches is to mitigate burst release of peptide that may otherwise occur with encapsulated approaches. Further, for surface‐conjugated peptide, there is an opportunity for control over the density of antigen presentation, which can have downstream impacts on immune responses.^[^ ^143^ ^]^ NPs synthesized from PLG polymer conjugated to self‐antigen effectively induced tolerance in mouse models of T1D and RR‐EAE. Delivery of these nanoparticles increased T_REG_ expansion and IL‐10 cytokine production, while decreasing disease severity.^[^ ^144^, ^145^ ^]^ However, in some contexts the design choice of material and whether antigen is conjugated or encapsulated can drive differential outcomes. Comparing self‐antigen conjugated to the surface of polystyrene NPs to encapsulation in PLG NPs, it was found this impacted allergy treatment in mice.^[^ ^146^ ^]^ The authors found that each construct could prevent allergy induction, including reduction of OVA‐specific IgE (Figure 7D); however, the polystyrene conjugated to antigen triggered anaphylaxis—a strong and dangerous allergic response—in mice presensitized to an allergen (Figure 7E). PLG‐antigen conjugated particles did not drive anaphylaxis but were less effective at modifying T_H_2 responses than PLG particles encapsulating antigen. A potential reason for this suggested by the authors is that allergic responses are mediated by IgE molecules recognizing antigen on the particle surface. Thus, antigen bound to the surface of NPs is potentially able to bind to IgE, driving an allergic response before particles are able to promote changes in APCs and T cells. This highlights how even where antigen is located within a material should be informed by biological circumstance.

### 6.4 Encapsulating Antigen Alone within Particles Can Effectively Drive Tolerance

One final approach to discuss is the use of nano and MPs that encapsulate antigen without tolerizing cue or ligand with the end goal of controlled release. These approaches have the advantage of being relatively straightforward while allowing for sustained delivery of an antigen of interest. However, the trade‐off for this less complex approach is reduced precise control over which other pathways are manipulated in coordination with antigen delivery. Like other particle delivery methods, this approach can increase delivery to APCs and enable control over trafficking dynamics through size selection. Multiple groups have developed carboxylated PLG polymer NPs loaded with antigens relevant to autoimmunity including T1D, EAE, and model antigens.^[^ ^147^, ^148^, ^149^, ^150^ ^]^ Over several published studies, the authors have demonstrated that these NPs are able to induce T_REG_ induction and protect against disease in an IL‐10 and T_REG_ dependent manner. In recent work, the authors also demonstrated that these particle constructs modulated CD8 responses as well—highlighting the multipronged nature of induced tolerance. In a recent study, the authors showed that changing the molecular weight and polymer composition of antigen NPs could have strong effects on disease outcomes in the context of EAE.^[^ ^151^ ^]^ Specifically, they demonstrated that low dose PLA NPs were more efficacious than PLG particles at disease mitigation (Figure 7F) and that PLA particles more strongly associated with liver sinusoidal cells—a cell population that is inherently tolerogenic. Many other groups have used PLG nano and microscale particles to successfully deliver antigen in mouse models for T1D, EAE, and skin transplant.^[^ ^152^, ^153^, ^154^, ^155^, ^156^ ^]^ In an approach for allergy, virus‐like particles were used to deliver antigens.^[^ ^157^ ^]^ The authors tested whether external or internal antigen display by the virus‐like particle affected allergen outcomes and found that encapsulated peptide was protective against disease. As in the allergy example in the previous section,^[^ ^146^ ^]^ it seems that encapsulation is a more effective and safer route for allergy delivery than surface conjugation.

Another encapsulation approach is to use liposomes. Liposomes are lipid‐based particles composed of a bilayer of lipid similar to a cell membrane. The structure enables delivery of hydrophobic cargo delivered in the lipophilic bilayer, as well as hydrophilic cargo in the inner aqueous space. Further, manipulation of lipid composition affects mechanical properties such as stiffness that can alter interactions with cells. As such, liposomal delivery of antigens and tolerizing vaccines has enabled more effective induction of tolerance over soluble methods. While previous examples in this review have used liposomes as a delivery method, the primary tolerizing effect was due to another delivered cue or ligand. In addition to previously discussed examples, manipulation of liposome stiffness has been used to alter tolerance induction. Building on previous work that linked stiffer particle composition with APC uptake and T cell activation, the authors of this study hypothesized that changing the stiffness of liposomes would similarly impact T_REG_ induction.^[^ ^158^ ^]^ Liposome rigidity was controlled by altering which lipids were in the membrane, then assayed using atomic force microscopy (Figure 7G). They demonstrated that more rigid liposomes were more effective at inducing T_REG_ responses (Figure 7H), which shows how important innate material properties can be for governing immune responses.

## 7 Clinical Development

While progress in the preclinical work described in the previous sections represents excellent progress in developing biomaterial approaches for antigen‐specific tolerance, it is important to also examine progress moving these advances into the clinic. The most prominent example of antigen‐specific tolerance in clinical use is specific immunotherapy (SIT) for allergies.^[^ ^159^ ^]^ SIT delivers allergens with/out adjuvant to shift allergenic immune responses based on T_H_2 responses toward alternative T cell responses such as T_REG_. While SIT is an encouraging example of clinical antigen‐specific tolerance, it requires weekly to monthly injections for years to achieve alleviation of symptoms and is effective mainly in mild allergies. Delivery of soluble antigen alone has been demonstrated to be largely ineffective for generating antigen‐specific tolerance in cases of autoimmunity and could even be dangerous for more severe allergies such as peanut allergies. To address the shortcomings of SIT, clinical trials that modify codelivered components, delivery route, and dose have been explored to increase the potency of this therapy.^[^ ^160^, ^161^ ^]^

Antigen therapy has also been explored for autoimmunity in the clinic. These therapies typically deliver soluble antigen or soluble antigen and another cue to patients via a multitude of delivery routes. This has been tested extensively in T1D, in large part due to knowledge of self‐antigens targeted during disease. However, despite preclinical success of antigen therapies in mouse models such as the NOD mouse, none of these therapies have successfully produced durable tolerance in patients.^[^ ^162^, ^163^ ^]^ The same is true for antigen therapies in MS.^[^ ^164^ ^]^ The lack of translatability of soluble antigen therapies highlights both crucial differences between human and murine models as well as the need for potentially more advanced delivery approaches. Thus, the benefits of materials described in this review may offer a way to increase the potency of therapeutics in a meaningful way for patients.

Despite preclinical success of many materials approaches for antigen‐specific therapy many of these approaches have not yet translated into clinical trials. This could be in part due to the lack of documented antigen‐specific tolerance to be achieved in human patients for autoimmunity by any approach as well as the stage of development of many antigen‐specific material approaches, or even the presence of an already crowded disease‐modifying therapy market in diseases such as MS, requiring a high burden of efficacy for a return on investment. Excitingly, one group has brought their NP delivery system into clinical trials for Celiac Disease, wherein gluten is targeted by the immune system.^[^ ^165^ ^]^ While an early trial, the particles were able to reduce interferon‐producing cells in treated patients compared to baseline in response to antigen. Additionally, Selecta Biosciences has completed multiple phase II clinical trials and recruiting phase III trials using rapamycin NPs to prevent development of antidrug antibodies.^[^ ^166^, ^167^ ^]^ This is an example of the potential for tolerance to be a mechanism not just for autoimmunity, but to enhance the efficacy of other approaches. While there are few published trials in materials for antigen‐specific tolerance, there are several companies developing phase I clinical trials based on technologies described in this review. Parvus Therapeutics is developing peptide‐MHC particles for the clinic for a variety of autoimmune applications, with phase I trials targeted for 2023. In addition to the previous trials discussed, Selecta is also working to develop their NP platform for other applications, including tolerance in primary biliary cholangitis.

## 8 Conclusions and Outlook

Allergy and autoimmunity represent a large clinical burden in today's biomedical landscape. Therapies that induce antigen‐specific immune tolerance offer a potential opportunity to change the way these diseases are treated in patients through selectively modifying immune responses toward disease antigens, while preserving healthy immune function. Biomaterials offer the tunable properties that may be required to orchestrate the complex interactions of signals to promote tolerance effectively in a clinical setting. This review covered the various strategies available used to promote tolerance using materials, including codelivery of small molecule drugs and metabolites, delivery of more targeted ligands and cytokines, modifying the material properties and immunogenicity of antigens themselves, targeted delivery of antigen to tolerogenic tissues and cells, and the innate modulatory property of delivering antigen in a material context. While each of these approaches have advantages and disadvantages, results have demonstrated that there is unlikely to be a one‐size‐fits‐all solution between antigen therapy and biomaterials due to the heterogeneity of disease. A polymer NP encapsulating antigen may promote tolerance in one animal model, while having little to no effect in another. A larger material may serve as a drug depot to recruit tolerogenic cells, while smaller materials may more effectively drain to immunological tissues such as lymph nodes. While the breadth of current work on materials is exciting, there is a need for more advanced analysis of immune responses in these delivery methods. Approaches such as single‐cell sequencing, which have become mainstays of the immunological research, could provide new insight into how biomaterial‐delivered antigens alter immune cells in a nonbiased manner. Further, for these approaches to have clinical viability, the long‐term durability of treatments needs to be tested more strenuously, as well as more critical evaluation of the selectivity of these approaches. Finally, for patients to receive the potential benefits of antigen‐specific therapies delivered via biomaterials, there needs to be a greater push to develop clinical programs to test therapies in patients.

## Conflict of Interest

C.M.J. is an employee of the VA Maryland Health Care System. The view reported here do not reflect those of the United States Government or Department of Veterans Affairs. C.M.J. has an equity position with Vaccitech plc. The remaining authors declare no commercial or financial relationships that could be construed as a potential conflict of interest.
